# The Emergence of Chromosomally Located *bla*_CTX-M-55_ in *Salmonella* From Foodborne Animals in China

**DOI:** 10.3389/fmicb.2019.01268

**Published:** 2019-06-05

**Authors:** Chuan-Zhen Zhang, Xiao-Min Ding, Xiao-Ling Lin, Ruan-Yang Sun, Yue-Wei Lu, Run-Mao Cai, Mark A. Webber, Huan-Zhong Ding, Hong-Xia Jiang

**Affiliations:** ^1^National Risk Assessment Laboratory for Antimicrobial Resistance of Animal Original Bacteria, College of Veterinary Medicine, South China Agricultural University, Guangzhou, China; ^2^Guangdong Key Laboratory for Veterinary Drug Development and Safety Evaluation, College of Veterinary Medicine, South China Agricultural University, Guangzhou, China; ^3^Quadram Institute Bioscience, Norwich, United Kingdom

**Keywords:** chromosomal, *bla*_CTX-M-55_, *Salmonella*, Indiana, transfer

## Abstract

The emergence and increase in prevalence of resistance to cephalosporins amongst isolates of *Salmonella* from food animals imposes a public health threat. The aim of the present study was to investigate the prevalence and characteristics of CTX-M-producing *Salmonella* isolates from raw meat and food animals. 27 of 152 (17.76%) *Salmonella* isolates were ESBL-positive including 21/70 (30%) from food animals and 6/82 (7.32%) from raw meat. CTX-M-55 was the most prevalent ESBL type observed (12/27, 44.44%). 7 of 12 CTX-M-55-positive *Salmonella* isolates were *Salmonella* Indiana, 2 were *Salmonella* Typhimurium, 2 were *Salmonella* Chester, and the remaining isolate was not typeable. Eight CTX-M-55-positive *Salmonella* isolates were highly resistant to fluoroquinolones (MIC_CIP_ = 64 ug/mL) and co-harbored *aac(6’)-Ib-cr* and *oqxAB*. Most of the CTX-M-55 positive isolates (11/12) carried *bla*_CTX-M-55_ genes on the chromosome, with the remaining isolate carrying this gene on a transferable 280 kb IncHI2 plasmid. A chromosomal *bla*_CTX-M-55_ gene from one isolate transferred onto a 250 kb IncHI2 plasmid which was subsequently conjugated into recipient strain J53. PFGE and MLST profiles showed a wide range of strain types were carrying *bla*_CTX-M-55_. Our study demonstrates the emergence and prevalence of foodborne *Salmonella* harboring a chromosomally located *bla*_CTX-M-55_ in China. The co-existence of PMQR genes with *bla*_CTX-M-55_ in *Salmonella* isolates suggests co-selection and dissemination of resistance to both fluoroquinolones and cephalosporins in *Salmonella* via the food chain in China represents a public health concern.

## Introduction

*Salmonella* species are the second most common bacterial cause of foodborne gastroenteritis worldwide and almost 80.3 million foodborne illness per year are caused by non-typhoid *Salmonella* infections (Majowicz et al., 2010). Extended-spectrum cephalosporins (ESCs) are effective drugs of choice in children for treatment of non-typhoid salmonellosis, due to the contraindication for use in children of fluoroquinolones (FQs), the classical first-line antibiotics. The emergence of *Salmonella* isolates resistant to ESC is a worldwide public health concern (Arlet et al., 2006). Resistance to these drugs is mainly mediated by the bacterial production of extended-spectrum β-lactamases (ESBLs) with CTX-M-type enzymes being the most common.

CTX-M genes have successfully disseminated globally and are common in clinical settings, communities, livestock and companion animals. There are many CTX-M variants of which, CTX-M-15 and CTX-M-14 are the most prevalent (Zhao and Hu, 2013; de Jong et al., 2014). However, the epidemiology of CTX-M-type ESBLs is evolving rapidly. A number of minor allelic variants have been described and classified as belonging to one of six groups (CTX-M-1, CTX-M-2, CTX-M-8, CTX-M-9, CTX-M-25 and KLUC, named after the archetypal enzymes of each group) that differ from each other by ≥ 10% amino acid residues (D’Andrea et al., 2013).

CTX-M-55 is a CTX-M-15 variant that contains a substitution of A80V within the β-lactamase possessing enhanced cephalosporin-hydrolyzing activity (He et al., 2015) and has been detected as increasing rapidly in prevalence, especially in *Escherichia coli* from animals (Zheng et al., 2012; Cunha et al., 2017; Norizuki et al., 2018). Whilst the *bla*_CTX-M-55_ gene is less commonly detected in *Salmonella* from animals or humans, the first report was from human isolates in the United States and China in 2011 (Sjolund-Karlsson et al., 2011; Yu et al., 2011). Since then CTX-M-55 producing *Salmonella* have been identified from a number of different serotypes from Switzerland (Gallati et al., 2013), Japan (Imoto et al., 2014), China (Wong et al., 2015), Korea (Kim et al., 2017), Denmark (Torpdahl et al., 2017), and Thailand (Luk-In et al., 2018). CTX-M-55 carrying isolates from animals have been isolated from fish, pork and chicken (Nguyen et al., 2016; Nadimpalli et al., 2018). Since CTX-M-55 *Salmonella* isolates are increasingly detected and show high-level resistance to ESCs and are often cross-resistant to FQs, these *Salmonella* strains represent a potentially severe clinical and food safety issues and this warrants investigation of the prevalence of *bla*_CTX-M-55_-harboring *Salmonella*.

Carriage of CTX-M genes is mostly associated with a diverse set of transmissible plasmids (Canton et al., 2012). However, a small number of chromosomal CTX-M genes have been identified in several studies in *E. coli* where transfer into the chromosome was mediated by transposons or insertion sequences (Fabre et al., 2009; Hamamoto and Hirai, 2018).

In the present study, we investigated the prevalence of CTX-M-type ESBL-producing *Salmonella* isolates from food animals and raw meat in Guangdong province during 2015 and 2017, analyzed the characteristics of these CTX-M-55-positive strains including phenotypes, genotypes, genetic relatedness, and plasmid profiles.

## Materials and Methods

### Sample Collection and *Salmonella* Isolation, Identification

A total of 891 specimens were collected from the Guangdong province of China between 2015 and 2017. Of these samples, 453 fecal swabs from free-range food animals (84 from chickens, 249 from ducks, 107 from pigs, and 13 from geese) were obtained from veterinary clinics. 438 raw meat samples (156 from chicken meat, 35 from duck meat, and 247 from pork) were collected from different supermarkets. Fecal swabs and meat samples (cut into pieces) were placed into sterile selenite cysteine broth and incubated for 24 h at 37°C. Aliquots were then streaked on chromogenic medium selective for *Salmonella* (CHROMagar Microbiology, France) and incubated for another 24 h at 37°C. One purple colony was selected from each plate and then confirmed using the API20E system (bioMérieux, Marcy L’Étoile, France) and identified by MALDI-TOF MS (Axima-Assurance-Shimadzu). All isolates identified as *Salmonella* were stored at -80°C in Luria-Bertani (LB) broth containing 30% glycerol.

### Antimicrobial Susceptibility Testing, Detection of ESBL Genes and ESBL Production Verified by Phenotype

The minimum inhibitory concentrations (MICs) of cefotaxime (CTX) and ciprofloxacin (CIP) were determined in triplicate for each bacterial strain using the agar dilution method on Mueller-Hinton agar plates according to the CLSI reference method (CLSI-M100-S26). *E. coli* ATCC 25922 was used as the quality control strain. *Salmonella* isolates showing resistance to cefotaxime (with MIC ≥ 4 ug/mL) were screened for the presence of the ESBL-genes *bla*_TEM_, *bla*_SHV_, *bla*_OXA_, *bla*_CTX-M_ and *bla*_CMY -2_ by PCR using the primers and conditions described previously (Jiang et al., 2012). Amplified PCR products were submitted to BGI Life Tech Co., Ltd. (Beijing, China) for DNA sequencing and the identity of specific β-lactamase genes were determined using the protein BLAST algorithm^1^. Double disk synergy tests were performed to further verify ESBL production by using a central amoxicillin/clavulanic acid (AMC) disk, 15 and 20 mm (center to center) separately away from cefotaxime disks. Synergy was interpreted by a clear-cut enlargement of the inhibition zone of CTX disk near the inhibition zone of AMC disk (Jarlier et al., 1988).

### Detection of PMQR Genes, Mutations Within Quinolone Resistance-Determining Region (QRDR) of Target Genes and Serotyping of CTX-M-55-Positive Isolates

The presence of PMQR genes *qnrA*, *qnrB*, *qnrC*, *qnrD*, *qnrS*, *aac(6’)-Ib-cr*, *qepA*, and *oqxAB* from *bla*_CTX-M-55_-positive isolates was also investigated by PCR using primers and conditions as previously described (Jiang et al., 2012). Mutations in QRDRs of the target genes *gyrA*, *gyrB*, *parC*, *parE* were confirmed by PCR and sequencing and their DNA sequences were compared with the *Salmonella* Typhimurium LT2 genome as a reference.

CTX-M-55-producing *Salmonella* isolates were serotyped using *Salmonella* specific O and H antigens (Statens Serum Institute, Denmark) by the slide agglutination test according to the Kauffmann-White scheme.

### Pulsed-Field Gel Electrophoresis (PFGE) and Multilocus Sequence Typing (MLST)

Genetic relatedness of all *bla*_CTX-M-55_-harboring isolates were analyzed by pulsed-field gel electrophoresis (PFGE) of XbaI-digested genomic DNA using a CHEF-MAPPER System (BioRad Laboratories, Hercules, CA, United States) as previously described (Jiang et al., 2014). PFGE patterns were compared using the Dice similarity coefficient with BioNumerics software (Applied Maths, Sint-Martens-Latem, Belgium).

MLST was carried out by PCR and DNA sequence analysis of 7 housekeeping genes *aroC*, *dnaN*, *hemD*, *hisD*, *purE*, *sucA*, and *thrA* to determine the allelic profiles using software available at http://mlst.warwick.ac.uk/mlst/dbs/Senterica.

### Conjugation Experiments and Plasmid Analysis

Conjugation experiments of *bla*_CTX-M-55_ gene positive *Salmonella* isolates were conducted by liquid mating in LB broth using sodium azide-resistant *E. coli* J53 as the recipient strain. Transconjugants were selected on MacConkey agar containing cefotaxime (2 ug/mL) and sodium azide (300 ug/mL). The presence of *bla*_CTX-M-55_ in transconjugants was verified by PCR and sequencing as described. PFGE analysis was conducted using S1 nuclease (Takara Biotechnology, Dalian, China) digested genomic DNA as previously described (Barton et al., 1995) to identify the genetic location of *bla*_CTX-M_ genes. Primers used for *bla*_CTX-M_-probes were the same as those used to amplify CTX-M encoding genes. The resulting gels were analyzed by Southern blotting after transfer to Hybond-N+ membranes (GE Healthcare, Little Chalfont, United Kingdom) and probing with a DIG-labeled *bla*_CTX-M_ gene fragment according to the manufacturer’s instructions (DIG High Prime DNA Labeling and Detection Starter Kit I, Roche Applied Science, Mannheim, Germany). Restriction fragments of agarose-embedded DNA of strain H9812 digested with XbaI(Takara) at 37°C for 4 h was used as DNA size marker during electrophoresis.

### Whole Genome Sequencing

To characterize the genetic context of *bla*_CTX-M_ genes DNA from selected isolates was used to sequence whole genomic content. This was done by MajorBio Co., Shanghai, China. The resulting reads were trimmed and genomes assembled using “SPAdes v 3.11.0” (Nurk et al., 2013), annotated using “Prokka v 1.13” (Seemann, 2014) and mapped reads against reference genomes using “Bowtie2 v 2.3.4.3” (Langmead and Salzberg, 2012). Annotations and alignments were visualized in “Artemis.”

## Results

### *Salmonella* Isolation and Antimicrobial Susceptibility Phenotypes

We collected 891 samples for this study and 152 *Salmonella* were identified by MALDI-TOF MS of which, 46.05% (*n* = 70) were isolated from animals (13 from chickens, 43 from ducks, 11 from pigs, and 3 from geese). The remaining 82 samples came from raw meat (including 37 chicken meat, 5 duck meat, and 40 pork supermarket samples). The isolation rate of *Salmonella* strains from raw meat (17.90%) was similar to that from the farm animals (15.45%).

We also examined susceptibility of the 152 *Salmonella* isolates to cefotaxime and ciprofloxacin. Resistance rates to cefotaxime and ciprofloxacin were 26.97% (*n* = 41) and 30.26% (*n* = 46), respectively. In the 70 *Salmonella* isolates from animals, 30 displayed cefotaxime resistance (42.86%) and 34 ciprofloxacin resistant (48.57%). The 82 *Salmonella* isolates from raw meat, contained 11 isolates resistant to cefotaxime (13.43%) and 12 resistant to ciprofloxacin (14.63%). The rate of cross-resistant to both antibiotics were significantly higher for the animal isolates (32.86%) than for raw meat (4.88%).

### ESBL Characterization and Production, Serotyping of CTX-M-55-Producing Isolates

A total of 27 CTX-M ESBLs producing isolates were confirmed among the *Salmonella* isolates. Of these, 21/70 (30%) were from animals and 6/82 (7.32%) were from meat. We found that 12/27 (44.44%) were CTX-M-1 group members and all were confirmed as being *bla*_CTX-M-55_. There were also 12/27 that belonged to the CTX-M-9 group and were assigned as *bla*_CTX-M-27_ (*n* = 8), *bla*_CTX-M-14_ (*n* = 2) and *bla*_CTX-M-65_ (*n* = 2). The remaining 3 CTX-M-encoding genes were all identified as the hybrid allele *bla*_CTX-M-64_. We also found that 14/41 cefotaxime-resistant isolates were CTX-M negative.

Of the *bla*_CTX-M-55_ positive isolates, 10/12 were from animals and the remaining 2 were from meat samples. A clear-cut extension of the edge of the inhibition zone of CTX disk toward the AMC disk was seen from each *bla*_CTX-M-55_ carrying *Salmonella* strain, consistent with ESBL production. These isolates were also serotyped and 7 were *S.* Indiana, 2 *S.* Typhimurium, 2 *S.* Chester and 1 was untypeable (Table 1).

**Table 1:** Characteristics of *bla*_CTX-M-55_ gene-harboring *Salmonella* isolates.

Strains	Serotype	Year	Sources	Cephalosporins resistance characterization			Quinolone resistance characterization					
				MIC_CTX_	β-lactamase genes	*bla*_CTX-M-55_ location	MIC_CIP_	PMQR genes	Mutations in GyrA/GyrB	Mutations in ParC/ParE	MLST
HZP3	Typhimurium	2016	pork	128	*bla*_CTX-M-55_ *bla*_TEM-1_	chromosome	1	*oqxAB*+*qnrS*	–/–	–/–	ND
LWP4	Typhimurium	2016	pork	128	*bla*_CTX-M-55_ *bla*_TEM-1_ *bla*_OXA-1_	chromosome	0.5	*qnrS*	–/–	–/–	ST34
OJM1	Indiana	2017	Chicken meat	256	*bla*_CTX-M-55_ *bla*_OXA-1_	chromosome	64	*aac(6’)-Ib-cr*+*oqxAB*	S83F D87N/–	T57S S80R/–	ST17
PJM1	ND	2017	Chicken meat	128	*bla*_CTX-M-55_ *bla*_TEM-1_ *bla*_OXA-1_	chromosome	4	*qnrS*	–/–	T57S/–	ST321
OYM4	Indiana	2017	duck	256	*bla*_CTX-M-55_ *bla*_OXA-1_	chromosome	64	*aac(6’)-Ib-cr*+*oqxAB*	S83F D87N/–	T57S S80R/–	ST17
OYM6	Indiana	2017	duck	256	*bla*_CTX-M-55_ *bla*_OXA-1_	chromosome	64	*aac(6’)-Ib-cr*+*oqxAB*	S83F D87N/–	T57S S80R/–	ST17
OYM8	Indiana	2017	duck	256	*bla*_CTX-M-55_ *bla*_OXA-1_	chromosome	64	*aac(6’)-Ib-cr*+*oqxAB*	S83F D87N/–	T57S S80R/–	ST17
OYM9	Indiana	2017	duck	256	*bla*_CTX-M-55_ *bla*_OXA-1_	chromosome	64	*aac(6’)-Ib-cr*+*oqxAB*	S83F D87N/–	T57S S80R/–	ST17
OYM10	Chester	2017	duck	256	*bla*_CTX-M-55_ *bla*_OXA-1_	chromosome	64	–	D87N/–	T57S S80R/–	ST343
OYM13	Indiana	2017	duck	256	*bla*_CTX-M-55_ *bla*_OXA-1_	chromosome	64	*aac(6’)-Ib-cr*+*oqxAB*	S83F D87N/–	T57S S80R/–	ST17
OYZ3	Indiana	2017	duck	256	*bla*_CTX-M-55_ *bla*_OXA-1_	chromosome	64	*aac(6’)-Ib-cr*+*oqxAB*	S83F D87N/–	T57S S80R/–	ST17
OYZ4	Chester	2017	duck	128	*bla*_CTX-M-55_ *bla*_TEM-1_ *bla*_OXA-1_	plasmid	4	*qnrS*	–/–	–/–	ST27


*ND, not determined.*

### Detection of Ciprofloxacin Resistance Mechanisms in CTX-M-55-Producing Isolates

Multiple QRDR mutations in *gyrA* and *parC* were detected in the high-level quinolone resistant isolates that co-harbored *aac(6’)-Ib-cr* and *oqxAB*. However, strains with an MIC_CIP_ in the 0.5 to 4 ug/mL range did not contain mutations in the QRDRs of target genes, but all contained *qnrS* (Table 1).

The 12 CTX-M-55 producers were all ciprofloxacin resistant and 8 exhibited high-level resistance (MIC_CIP_ = 64 ug/mL), 4 isolates demonstrated lower resistance (MIC_CIP_ = 0.5–4 ug/mL). For PMQR determinants, 11 of 12 *bla*_CTX-M-55_-positive isolates were found to harbor at least one PMQR gene. The combination of *aac(6’)* - *Ib-cr* + *oqxAB* (*n* = 7) dominated followed by *qnrS* (*n* = 3) and *oqxAB + qnrS* (*n* = 1). One isolate completely lacked any PMQR genes (Table 1).

### Genetic Relatedness and Molecular Typing Analysis of CTX-M-55-Producing Isolates

**FIGURE 1:**
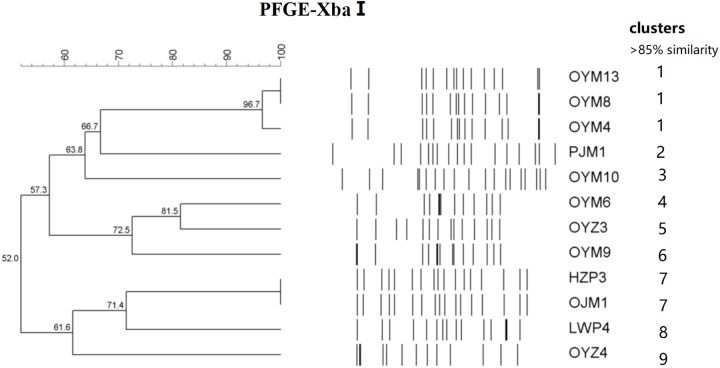
PFGE fingerprinting patterns and other characteristics of *Salmonella* isolates harboring *bla*_CTX-M-55_ genes.

The 12 CTX-M-55 isolates produced 11 different profiles that were divided into 9 different PFGE clusters designated 1–9 with 85% genetic similarity. Five MLST profiles were determined including ST17, ST27, ST34, ST321, and ST343. ST17 was most prevalent (*n* = 7, 58.3%), ST27, ST34, ST321, and ST343 were represented by one isolate each and no ST could be determined for one isolate (Figure 1).

### *bla*_CTX-M-55_ Hybridization and Plasmid Analysis

**FIGURE 2:**
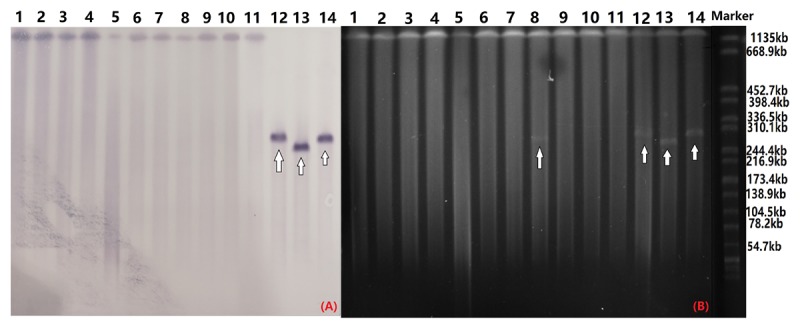
Southern blot hybridization with the *bla*_CTX-M-55_ probe **(A)** and S1-PFGE of S1 nuclease-digested genomic DNA **(B)**. The arrow indicates plasmids in the isolates. OYM4 (lane 1), OYM6 (lane 2), OYM8 (lane 3), OYM9 (lane 4), OYM13 (lane 5), OYZ3 (lane 6), OJM1 (lane 7), HZP3 (lane 8), LWP4 (lane 9), OYM10 (lane 10), PJM1 (lane 11), OYZ4 (lane 12), C-PJM1 (lane 13), C-OYZ4 (lane 14), Marker (H9812).

S1-PFGE and southern hybridization analysis of all 12 *bla*_CTX-M-55_-positive *Salmonella* isolates and 2 transconjugants found that *bla*_CTX-M-55_ was chromosomally located in 11 of the isolates and in one isolate was present on a 280 kb plasmid (Figure 2). Interestingly, we successfully obtained one transconjugant from PJM1 whose *bla*_CTX-M-55_ gene was chromosomal. This transconjugant carried the *bla*_CTX-M-55_ gene on a 250 kb IncHI2 plasmid suggesting a mechanism where transfer from the chromosome onto a plasmid was followed by conjugation of this plasmid, now carrying the resistance gene (Figure 2). Two narrow-spectrum β-lactamase gene, *bla*_TEM-1_ and *bla*_OXA-1_ and PMQR gene *qnrS* also co-transferred with *bla*_CTX-M-55_ in the transconjugants (Table 2). Analysis of the whole genome assembly of PJM1 identified the *bla*_CTX-M-55_ present within an 11 kb contig with 100% identify to plasmid pCSFA1096, previously identified from *Salmonella* in China. However, mapping of all reads from the total genome sequencing of PJM1 against the pCSFA1096 genome identified alignments only over the elements carrying the resistance genes and the rest of the plasmid backbone was not present. This suggests the *bla*_CTX-M-55_ gene originally transferred into the chromosome of PJM1 within a larger mobile element which retains the capacity to be mobilized. This supports our proposed model of transfer of this chromosomal element from PJM1 onto a plasmid and subsequent onward transmission.

**Table 2:** Characteristics of *bla*_CTX-M-55_ gene-positive transconjugants.

Transconjugants	Origin	Inc type	ESBL genes	PMQR genes	Plasmid size	Transfer rate
C-PJM1	chicken	IncHI2	*bla*_CTX-M-55_, *bla*_OXA-1_, *bla*_TEM-1_	*qnrS*	250 kb	58.9%
C-OYZ4	duck	IncHI2	*bla*_CTX-M-55_, *bla*_OXA-1_, *bla*_TEM-1_	*qnrS*	280 kb	74.4%


## Discussion

In the present study we found that 27 (65.85%) cefotaxime-resistant *Salmonella* strains produced CTX-M-type ESBLs. In this group, 21 were from food animals and 6 were from meat. The CTX-M-producing strains included 17 that were ciprofloxacin-resistant and 9 with decreased susceptibility to ciprofloxacin. In addition, at least one PMQR gene was detected in each of the 21 CTX-M isolates from animals and these results were similar to our previous study (Zhang et al., 2016). Together these studies suggest that the co-existence or co-transfer of PMQR genes in CTX-M-producing *Salmonella* strains increase their probability of survival in the presence of quinolones and/or cephalosporins (Liu et al., 2013).

The cefotaxime-resistant *Salmonella* strains included 5 CTX-M subtypes and CTX-M-55 was the most prevalent (*n* = 12). This was inconsistent with our previous studies that CTX-M-27 was the most prevalent ESBL in *Salmonella* strains isolated in 2009, 2010, and 2014 (Jiang et al., 2014; Zhang et al., 2016). In the current study, all the 12 CTX-M-55-producing strains were simultaneously non-susceptible to ciprofloxacin and almost all harbored PMQR genes. PMQR gene *qnrS* was only detected in low level ciprofloxacin resistant strains which contained no resistance-associated mutations in the QRDR. Though PMQR determinants only confer low level fluoroquinolone resistance, their existence (especially *qnr*) provide strains with a selective advantage under fluoroquinolones exposure and can accelerate the development of chromosome-mediated quinolone resistance (Robicsek et al., 2006; Strahilevitz et al., 2009).

Plasmids are key vectors in the global dissemination of antibiotic resistance genes in Gram-negative bacteria. Plasmid families including IncF, IncI1, IncI2, IncX, IncA/C, and IncHI2 play important roles in ESBL gene spread (Wang et al., 2018b). The *bla*_CTX-M-55_ genes are the second most abundant ESBL subtype in the Enterobacteriaceae (Zhang et al., 2014; Lupo et al., 2018). This is especially true of *E. coli* from both humans and animals in Asia and these are usually found on IncF and IncI1 plasmids (Zhang et al., 2014; Lupo et al., 2018; Wang et al., 2018a). Additional data from our laboratory presented evidence that the prevalence of *bla*_CTX-M-55_ in *E. coli* from both livestock and human origin is increasing. The F33:A-:B- and IncI1 plasmids have driven the spread of these genes in China. F33:A-:B- plasmids impart a significant biological advantage to their host and thus contribute to the increasing distribution of *bla*_CTX-M-55_ (Wang et al., 2018b).

In the present study, we found chromosomal copies of *bla*_CTX-M-55_ in 11/12 CTX-M-55-producing *Salmonella* strains. We speculate that the cross-species dissemination of *bla*_CTX-M-55_ from plasmids in *E. coli* to *Salmonella* chromosomes contributes to the spread and stable persistence of this gene in *Salmonella* (Wong et al., 2015). Following the first isolation from a food animal in 2010 in China (Yan et al., 2010), the detection rate of *S.* Indiana increase d rapidly, especially from veterinary clinics and food-producing animals. The highly fluoroquinolone and β-lactam-resistant *S.* Indiana ST17 is the most prevalent sequence type of this serovar in China. This may suggest an increasing disseminating trend of ST17 CTX-M-55-encoding *S.* Indiana. The highly drug-resistant *S.* Indiana ST17 is one of the most prevalent antimicrobial-resistant foodborne pathogens in China so that its isolation from animals is a public health concern (Wang et al., 2017; Zhao et al., 2017; Cao et al., 2018). Additionally, the monophasic variant of *S.* Typhimurium ST34 has already emerged in Europe and Asia (Arnott et al., 2018). A comparison of the genomes of a pork meat and a human isolate revealed only 10 single nucleotide polymorphisms (SNP). This indicated that human bacterium was acquired from pork meat (Arnott et al., 2018).

A major mechanism underpinning the global dissemination of β-lactam resistant bacteria is their possession of resistant plasmids with low fitness cost or stable carriage of ESBL genes in the chromosome although the later are currently uncommon. Before 2013, strains harboring chromosome-located ESBL encoding genes were sporadically detected in *Escherichia coli* (Garcia et al., 2005; Coque et al., 2008), *Salmonella* Concord (Fabre et al., 2009), *Klebsiella pneumoniae* (Coelho et al., 2010), *Proteus mirabilis* and *Morganella morganii* (Harada et al., 2012; Mahrouki et al., 2012). Since then, highly prevalent *E. coli* strains possessing chromosomal CTX-M-14 and CTX-M-15 β-lactamases were identified in 2013 (Hirai et al., 2013), 2016 (Hamamoto et al., 2016) and 2018 (Hamamoto and Hirai, 2018) separately. There may be a chromosomal *bla*_CTX-M_ transpositional unit responsible for the global dissemination of CTX-M-14 in *E. coli*. However, the nature and significance of the spread of chromosomally located *bla*_CTX-M_ genes remains unclear. We found most isolates carrying CTX-M-55 had the gene in a chromosomal context and these were from a diverse set of strain types. Transfer was however, possible from a strain (PJM1) with a chromosomal gene and the transconjugants carried the gene on a 280 kb IncHI2 plasmid. Together these data suggest that CTX-M-55 readily incorporates into the chromosome of *Salmonella* and that this is associated with PMQR carriage but that the element carrying the CTX-M-55 gene can move onward onto plasmids for further dissemination.

A recent study in Cambodia demonstrated that spread of CTX-M-55-type *S. enterica* from pork and fish samples was mediated by MDR IncA/C2 and IncHI2 plasmids (Nadimpalli et al., 2018). IncHI2 plasmids are the fifth most widely disseminated plasmid type that mediate transmission of antibiotic resistance genes. These are primarily found in *Salmonella*, *E. coli*, *Enterobacter cloacae* and *Klebsiella pneumoniae* of human and avian sources (Garcia Fernandez et al., 2007; Li et al., 2013; Haenni et al., 2016; Zhang et al., 2016).

## Conclusion

In conclusion, we identified foodborne *Salmonella* harboring chromosomally located *bla*_CTX-M-55_ from China. These strains are simultaneously non-susceptible to fluoroquinolones. The co-existence of PMQR genes and CTX-M ESBL genes indicated co-selection for these determinants which may accelerate the dissemination of multi-drug resistance. Importantly, these strains may promote the development of isolates resistant to both cefotaxime and ciprofloxacin. The determination of the mechanisms and dissemination routes of ESBL-producing *Salmonella* is critical for animal and human health and understanding the interplay between movement of resistance genes between plasmids and chromosomal locations is important to understand the dynamics and evolutionary consequences of spread of antimicrobial resistance (AMR). This would also provide useful information to effectively control the development of antibiotic resistance to cephalosporins and fluoroquinolones.

## Ethics Statement

This study protocol was approved by the South China Agriculture University Animal Ethics Committee. The strains of free-range food animal origin were isolated from fecal swabs of healthy chickens, pigs, ducks, and geese and the owners of the animals gave permission for their animals to be used in this study.

## Author Contributions

C-ZZ, H-ZD, and H-XJ conceived and designed the experiments. C-ZZ, X-MD, X-LL, R-YS, Y-WL, and R-MC performed the experiments. C-ZZ, MW, H-ZD, and H-XJ analyzed the data. C-ZZ, X-MD, X-LL, Y-WL, and R-MC contributed reagents, materials, and analysis tools. C-ZZ, MW, and H-XJ wrote the manuscript.

## Conflict of Interest Statement

The authors declare that the research was conducted in the absence of any commercial or financial relationships that could be construed as a potential conflict of interest.

## Footnotes

1 https://blast.ncbi.nlm.nih.gov
